# Long‐term postoperative pain evaluation in dogs with thoracolumbar intervertebral disk herniation after hemilaminectomy

**DOI:** 10.1111/jvim.15800

**Published:** 2020-05-28

**Authors:** Natalia Zidan, Julia Medland, Natasha Olby

**Affiliations:** ^1^ Department of Clinical Sciences College of Veterinary Medicine, North Carolina State University Raleigh North Carolina USA; ^2^ Comparative Medicine Institute North Carolina State University Raleigh North Carolina USA

**Keywords:** algometry, mechanical thresholds, neuropathic pain, postoperative pain, spinal cord injury

## Abstract

**Background:**

Chronic neuropathic pain is a common complication in people with spinal cord injury (SCI) but has not been investigated in dogs.

**Objective:**

To determine the reliability of measuring spinal mechanical sensory thresholds (MSTs) in dogs and to compare MSTs of healthy dogs and dogs with SCI caused by acute thoracolumbar intervertebral disk extrusion after hemilaminectomy over a 1‐year period.

**Study Design:**

Prospective study.

**Animals:**

Thirty‐two healthy and 40 SCI dogs.

**Methods:**

Dogs were divided into group 1 (healthy Dachshunds), group 2 (healthy dogs including several breeds), and SCI group. The MSTs were measured using algometry at an incision (thoracolumbar) and control site. Dogs in group 1 were tested once; those in group 2 were tested for 5 consecutive days; and SCI dogs were tested on days 7, 14, 28, 42, 180, and 365 postoperatively. The MSTs were compared among days in healthy and SCI dogs and between SCI and healthy dogs using mixed effect models. *P* < .05 was considered significant.

**Results:**

At the incision site of SCI dogs, MST was significantly lower than in healthy dogs for 42 days postoperatively, but not subsequently. However, 4/27 dogs had control site MST below the reference range 1 year after surgery.

**Conclusions and Clinical Importance:**

Mechanical sensory thresholds normalize by 6 months after surgery in most dogs with SCI. Approximately 15% of SCI dogs may develop chronic neuropathic pain. Improving long‐term pain assessment of SCI dogs is important for offering treatment options and advising owners.

**Abbreviations**
CTR: cutaneous trunci reflex
MFS: modified Frankel score
MST: mechanical sensory threshold
OFS: open field score
QOL: quality of life
SCI: spinal cord injury
SSMS: surgical site manipulation score
TL‐IVDE: thoracolumbar intervertebral disk extrusion

## 1 INTRODUCTION

Pain assessment is essential for effective patient care in veterinary medicine. According to the International Association for the Study of Pain (IASP), “pain is an unpleasant sensory and emotional experience, associated with actual or potential tissue damage,” and will impact the patient's quality of life (QOL).^1^ It can be classified as nociceptive or neuropathic. Nociceptive pain is caused by a noxious stimulus that is processed by a normally functioning somatosensory system as a physiologic response and has been studied extensively in veterinary patients.^2^, ^3^, ^4^, ^5^, ^6^, ^7^, ^8^ Neuropathic pain is an inappropriate response caused by damage or dysfunction of the somatosensory system, and human patients report burning, stabbing, numbness, and tingling sensations.^1^, ^3^, ^5^, ^6^, ^8^, ^9^, ^10^, ^11^, ^12^, ^13^, ^14^ It is likely to be more difficult to identify neuropathic pain in veterinary patients, but dogs with Chiari malformation and syringomyelia are thought to exhibit signs consistent with this type of pain.^15^, ^16^, ^17^

Approximately 50% to 80% of people with spinal cord injury (SCI) develop chronic neuropathic pain, and it is possible that the same is true in dogs after SCI.^4^, ^8^, ^18^ Intervertebral disk herniation is the most common cause of SCI in dogs, and successful outcome is based on neurological recovery. The possibility that affected dogs suffer chronic neuropathic pain that impacts the quality of their recovery has not been investigated because of the challenges associated with recognizing and quantifying pain, and in particular, neuropathic pain in pet dogs.

The use of subjective and objective measurements of pain is becoming more common in veterinary medicine, especially in research and clinical trials. Subjective methods of pain assessment in dogs include validated clinical metrology instruments that grade specific behaviors on ordinal scales. Some examples are the Glasgow short form composite pain score and the Colorado State University canine acute pain scale.^19^, ^20^ More objective methods such as quantitative sensory testing using a variety of different stimuli (mechanical, thermal, vibration) also have been used in dogs.^17^, ^21^, ^22^, ^23^, ^24^, ^25^, ^26^, ^27^, ^28^, ^29^, ^30^, ^31^, ^32^, ^33^ Pressure algometry is a technique used to establish mechanical sensory thresholds (MSTs). Gradually increasing pressure is applied to the area of interest until a response is obtained that indicates a noxious threshold has been reached, and studies have demonstrated its reliability and feasibility in dogs.^21^, ^24^, ^29^, ^33^

Our aims were to establish the range and reliability of measurement of paraspinal MSTs in healthy dogs, to measure paraspinal MSTs in dogs with SCI caused by acute thoracolumbar intervertebral disk extrusion (TL‐IVDE) surgically decompressed by hemilaminectomy, to describe the temporal recovery of paraspinal MSTs in SCI dogs, and finally to compare paraspinal MSTs in healthy dogs and dogs with SCI. We hypothesized that paraspinal MSTs would be lower in SCI dogs than in healthy dogs immediately after surgery and that although their MSTs would increase over time after surgery, a subset of dogs would have persistently low paraspinal MSTs because of the development of SCI‐associated neuropathic pain.

## 2 MATERIALS AND METHODS

### 2.1 Animals

This prospective study conducted from February 2016 until November 2017 recruited neurologically normal client‐owned dogs and dogs with acute SCI caused by TL‐IVDE. Dogs were assigned to 3 different groups: control group 1 consisting of healthy Dachshunds assessed on a single day by 2 observers to establish the reference range of paraspinal MSTs and interobserver reliability; control group 2 consisting of dogs that were tested on 5 consecutive days to assess test and retest consistency; and the SCI group consisting of dogs diagnosed with TL‐IVDE that had been surgically treated.

All dogs had to weigh <20 kg and be between 2 to 12 years of age. To be included in control groups, dogs had to have no previous history of thoracolumbar myelopathy or back pain and normal neurological and orthopedic examinations. Dachshunds were recruited for the single‐day assessment group because this breed has the highest risk of TL‐IVDE and represents 50% to 75% of dogs with acute TL‐IVDE treated surgically.^34^, ^35^, ^36^

Dogs in the SCI group had to have a ≤3‐day history of nonambulatory paraparesis or paraplegia with or without pain perception at time of presentation, have a diagnosis of acute TL‐IVDE with magnetic resonance imaging (1.5 T Siemens Symphony, Cary, North Carolina) or computed tomography (Siemens Perspective 64 slice, Cary, North Carolina) and be treated surgically by hemilaminectomy and fenestration. Decompressive surgery was performed within 24 hours of presentation. A hemilaminectomy was performed over sites of compression. All dogs had fenestration at the disk space that had extrusion in addition to prophylactic fenestration at T11‐12 through L2‐3 intervertebral disk spaces. Dogs in this group had participated in 1 of 2 clinical trials in which data were collected prospectively. All were in the control groups of these clinical trials and did not receive additional treatments that might alter their results.^37^, ^38^ Immediately after surgery, a standard postoperative analgesic protocol was instituted for all participants (Table 1). Exclusion criteria for all dogs in this study included comorbidity that would affect pain assessment (eg, clinically relevant orthopedic disease, other systemic disease such as diabetes mellitus), as well as intolerance of daily handling.

**TABLE 1:** Drugs used postoperatively for pain, anxiety control, and to facilitate bladder expression

Drug	Dose/route	Frequency	Duration	Reason
Hydromorphone^a^	0.05‐0.1 mg/kg IV	8 h	24 h	Pain control
Carprofen^b^ ^,^ ^c^	2.2 mg/kg PO	12 h	7 d	Pain control
Meloxicam^c^ ^,^ ^d^	0.1 mg/kg PO	24 h	7 d	Pain control
Fentanyl^e^	3–5 μg kg^−1^ h^−1^ Transdermal	Continuous release	5 d	Pain control
Gabapentin^f^	10 mg/kg PO	8 h	10 d	Pain control
Phenoxybenzamine^g^ ^,^ ^h^	0.5 mg/kg PO	12 h	As needed	Bladder expression
Prazosin^g^ ^,^ ^i^	1–2 mg/dog PO	8‐12 h	As needed	Bladder expression
Diazepam^j^	0.5 mg/kg PO	8 h 20 min before bladder expression	As needed	Bladder expression
Trazodone^k^	2–8 mg/kg PO	8‐12 hours	As needed	Anxiety

a Hydromorphone: West Ward Pharmaceutics, Eatontown, New Jersey.

b Carprofen: Rimadyl, Zoetis, Lincoln, Nebraska.

c The nonsteroidal anti‐inflammatory drug (NSAID) choice was influenced by which NSAID had been administered before referral. If dogs were receiving corticosteroids before admission, omeprazole (1 mg/kg, PO, q24h) and prednisone in a tapering course were substituted for the NSAID.

d Meloxicam: Metacam, Boehringer Ingelheim, St Joseph, Missouri.

e Fentanyl transdermal patch, Mylan, Morgantown, West Virginia.

f Gabapentin: Method, Fort Worth, Texas.

g Dogs were placed on phenoxybenzamine if they were unable to urinate. If their bladder could not be expressed readily after 48 hours, on this drug prazosin was substituted.

h Phenoxybenzamine: compounded by NCSU VH Pharmacy, Raleigh, North Carolina.

i Prazosin: Mylan, Morgantown, West Virginia.

j Diazepam: Mylan, Rockford, Illinois.

k Trazodone: TEVA, North Wales, Pennsylvania.

Owners of dogs that met the inclusion criteria for each group were informed of the study details and signed a consent form. All procedures were performed with approval of the North Carolina State University Institutional Animal Care and Use Committee.

### 2.2 Data collection

Physical and neurologic examinations were performed on all dogs by the same clinician (Natalia Zidan). Minimum data collected from all dogs included body weight, breed, sex, and age. During the neurologic examination, the cutaneous trunci reflex (CTR) also was recorded and categorized as normal or hyperesthetic. A hyperesthetic CTR was characterized by a strong reflex contraction of the cutaneous trunci muscle in response to light touch to the skin (Video S1). In addition, for the SCI dogs, severity of neurological signs was graded using the modified Frankel score (MFS)^39^ and the modified open field score (OFS) (ranging from 0 to 12) at presentation and at days 42 and 365 postoperatively.^40^ At each evaluation of SCI dogs, postoperative pain was assessed by the same clinician (Natalia Zidan) using a surgical site manipulation score (SSMS; Supporting Information Data S1),^41^ before measurement of MST. Assessments from days 7, 14, 28, 42, 180, and 365 postoperatively were collected for analysis. In addition, at follow‐up evaluations, owners were asked if dogs were showing any signs of pain at home.

### 2.3 Mechanical sensory threshold measurement

A handheld manual pressure algometer with a 1 cm^2^ round rubber disk attached to a pressure meter (Pain Test FPK 10 pressure algometer; Wagner Instruments, Greenwich, Connecticut) was used for the MST evaluations. Before testing, dogs were acclimated to the examiners in a quiet room, with minimal distractions for 10 minutes. After acclimation, algometry was performed with the dogs in a standing position on the floor with minimal restraint. The investigator placed an arm under the dog's abdomen to prevent healthy dogs from sitting and to give support to the nonambulatory dogs while measurements were being taken. The algometer was applied with the other hand and perpendicular to the anatomical site being evaluated.

Pressure algometry was performed at 2 different sites over the paraspinal musculature with pressure applied approximately 2 cm lateral to the dorsal spinal processes. Measurements were obtained first at the level of the first to third thoracic vertebrae, defined as the control site. Subsequently, measurements were performed at the thoracolumbar junction, between T10 and L2 (TL site for healthy dogs or the surgical incision site of SCI dogs that had a hemilaminectomy performed). The MST measurements were performed by the same observer (Natalia Zidan) for all dogs, and by 2 observers (Natalia Zidan and Julia Medland) in group 1 control dogs by applying increasing pressure until the dog showed a behavioral response such as moving away, looking around, vocalizing, or attempting to bite, or until the maximal force (11 lb) was reached. Because the rate of force application could influence threshold measurements, the operator practiced consistent application force before the study. Measurements were performed in triplicate at each site, with a 1‐minute interval between measurements, and the mean score was calculated. In control group 1, there was a rest period of 10 minutes between observers. In SCI dogs, measurements were performed in the morning before daily analgesic medications were administered. Mechanical sensory thresholds of the SCI dogs were assessed at days 7, 14, 28, 42, 180, and 365 postoperatively.

### 2.4 Statistical analysis

Summary data were generated on demographics including age, sex, breed, and were compared among groups. Continuous data were evaluated for normality of distribution using the Shapiro‐Wilk*W* test. Because all variables were nonnormally distributed, data were reported as median and range and a nonparametric Wilcoxon rank sum test was used to compare variables among groups.

Baseline paraspinal MSTs were established from the single‐day testing summary data on control and thoracolumbar (incision) site results in healthy Dachshund dogs (control group 1). The MSTs of control and thoracolumbar sites were compared using a mixed effect model in which anatomical site was included as a fixed effect and dog as a random effect. To assess interobserver variability, the MST measurements of 2 observers were modeled using analysis of variance in which observers and dog were included as a fixed effect. The intraclass correlation was reported as the Shrout‐Fleiss reliability single score.

Mixed effect models were generated and least squares means estimated and compared to evaluate: test‐retest reliability of daily pressure algometry in control group 2 (5 daily measurements), changes in paraspinal MSTs in the SCI group postoperatively, paraspinal MSTs in SCI group compared with control group 1, the relationship between the CTR and MST, and MSTs compared with OFS in SCI groups. Details of each analysis are provided in Supporting Information Data S2. The CTR category of SCI dogs was also compared with healthy dogs. The Fisher exact test was used to determine if CTR category was different between healthy and SCI dogs at days 42 and 365.

For all data analysis, *P* < .05 was considered statistically significant, and where indicated, adjusted *P* values (Tukey's methods) were reported. A commercial statistical software program was used for all analyses (SAS version 9.4, Cary, North Carolina).

## 3 RESULTS

### 3.1 Animals

Seventy‐two dogs were enrolled in the study: 22 in control group 1, 10 in control group 2, and 40 in the SCI group. All 22 dogs in group 1 were Dachshunds whereas in group 2 there were 7 Dachshunds and 1 each of American Cocker Spaniel, Corgi, and Toy Poodle. Of the 40 dogs in the SCI group, 23 were Dachshunds, 8 were mixed‐breed dogs, 2 were Havanese, 2 were American Cocker Spaniels, and there was 1 each of Bichon Frise, Chihuahua, Corgi, French bulldog, and Shih Tzu. Median age at admission for all dogs was 6 years (range 2‐12 years). Age, sex, and body weight of the participating dogs did not differ significantly among groups (Table 2). At presentation, of the 40 dogs in the SCI group, severity of signs in 19 dogs was MFS grade 3; 13 dogs were grade 4; and 8 dogs were grade 5. No patient was excluded because of intolerance to algometry.

**TABLE 2:** Signalment characteristics of dogs in groups 1 and 2 (control groups) and SCI dogs

	Group 1 (n = 10)	Group 2 (n = 22)	SCI dogs (n = 40)	
Age (y) median (range)	8 (2‐12)	7 (2‐11)	5 (2‐11)	*P* = .27
Sex				*P* = .83
F	1	0	2	
FS	4	12	17	
M	0	1	3	
MC	5	9	18	
BW (kg) median (range)	5 (3.6‐8.6)	5.8 (4.1‐8)	6.6 (2.8‐14.5)	*P* = .07

Abbreviations: BW, body weight; SCI, spinal cord injury group.

### 3.2 Mechanical sensory thresholds

Minimal restraint was required to perform MST in control and SCI dogs. One control dog did not want to be held, but tolerated the test well.

#### 3.2.1 Control group 1: Healthy Dachshunds

**FIGURE 1:**
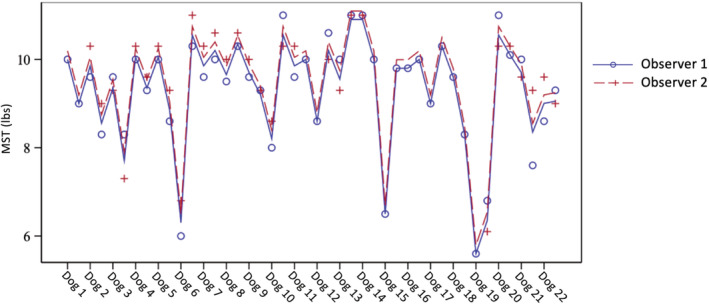
Interaction plot for MST in healthy Dachshunds (group 1) between two observers. Agreement between observers was excellent (κ = 0.91). MST, mechanical sensory threshold

In healthy Dachshunds tested once, the mean paraspinal MSTs of the control site and thoracolumbar site were 9.95 ± 0.68 lbs and 8.65 ± 1.41 lbs, respectively, with the thoracolumbar site MST being significantly lower than the control site (*P* < .001). Agreement between observers was excellent (κ = 0.91; Figure 1).

#### 3.2.2 Group 2: Test‐retest reliability

When testing control group 2 dogs once a day for 5 days, no significant interaction between algometry site and measurement day was identified (*P* = .81), indicating that no significant change in algometry measurements occurred over time and that the dogs tolerated repeated measurements.

#### 3.2.3 Recovery of MST after SCI and surgery

**FIGURE 2:**
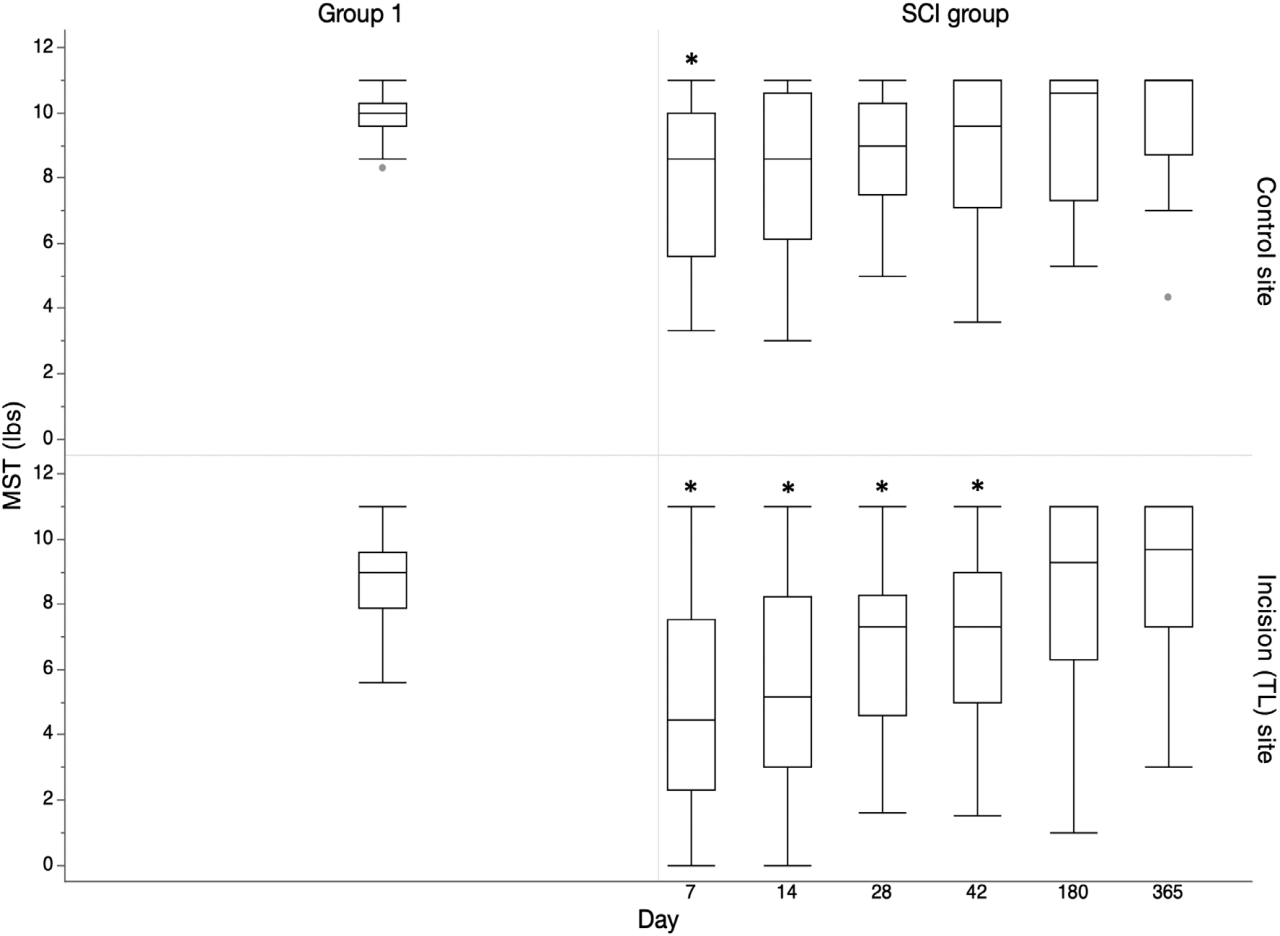
Box and whisker plot showing comparison between control and incision (thoracolumbar) sites among group 1 and SCI dogs. Mechanical sensory threshold of SCI dogs were significantly lower 7 days postoperatively at the control site and on days 7, 14, 28, and 42 after surgery at the incision site when compared with group 1 dogs (*P* < .001*). MST, mechanical sensory threshold; SCI, spinal cord injury; TL, thoracolumbar

All 40 dogs were evaluated on days 7 and 14. On days 28 and 42, 39 dogs were examined and at 6 months and 1 year postoperatively, 31 and 27 dogs, respectively, were evaluated with the remaining dogs lost to follow‐up. The MSTs of group 1 dogs and SCI dogs evaluated at each time point are shown in Figure 2. Mechanical sensory thresholds were decreased at both control and incision sites at 7 days after surgery, but increased over time. Mechanical sensory thresholds at the incision were significantly lower than at the control site (*P* < .001) until 6 months (Figure 2 and Supporting Information Data S3). When compared with group 1 dogs, MSTs in SCI dogs at the control site were significantly lower on day 7 (*P* = .01) and after that time had a wider range than did group 1 dogs, but were not significantly different (Figure 2). By contrast, MSTs at the incision site were significantly lower in SCI dogs on days 7, 14, 28, and 42 after surgery when compared with group 1 dogs (*P* < .001; *P* < .001; *P* = .001; *P* = .002; Figure 2). Similar to the control site, the range of MSTs was wider at the incision site at all time points evaluated when compared with group 1 dogs. To identify dogs that appeared to have lower thresholds than expected in the long term, we counted the number of dogs with MSTs below the minimum recorded in group 1 dogs at the control site (minimum of 8.3 lbs) and incision site (minimum of 5.6 lbs). At the incision site, 7/31 dogs were below the minimum at 6 months after surgery and 2/27 were below the minimum at 1 year after surgery. At the control site, 10/31 dogs were below the minimum at 6 months and 4/27 dogs were below the minimum at 1 year. Three of the dogs with low MSTs at 6 months were lost to follow‐up at 1 year.

### 3.3 Cutaneous trunci reflex and spinal palpation

**FIGURE 3:**
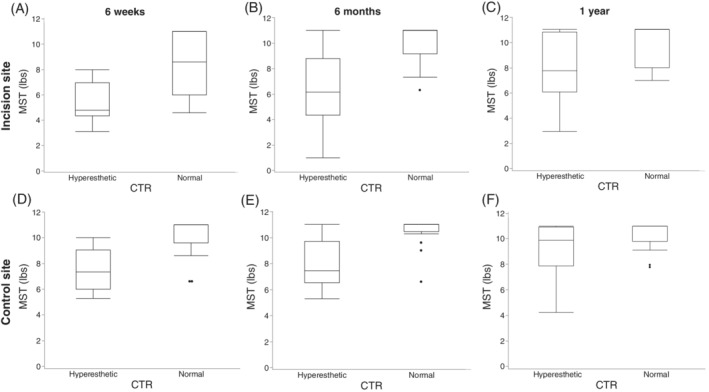
Box and whisker plot of MST values in SCI dogs grouped according to CTR category at 6 weeks, 6 months, and 1 year postoperatively. Top graphs (A, B, and C) represent MST at incision site, bottom graphs (D, E, and F) MST at control site. There is a significant correlation between the CTR categories and the MST values at both time points (*P* < .001). CTR, cutaneous trunci reflex; MST, mechanical sensory threshold; SCI, spinal cord injury

The CTR was evaluated in dogs of all 3 groups. Three dogs (3/22, 14%) in control group 1 and 2 dogs (2/10) in control group 2 had a CTR classified as hyperesthetic, resulting in a total of 5/32 (15.6%) control dogs with a hyperesthetic CTR. In the SCI group, CTR was evaluated in 35/40 dogs, 31/40 dogs, and 27/40 dogs at 6 weeks, 6 months, and at 1 year after surgery, respectively. Twelve dogs (12/53; 34%) had a hyperesthetic CTR at 6 weeks after surgery. At the 6‐month and 1‐year postoperative reevaluations, 14/31 (45%) dogs and 12/27 (44%) dogs had a hyperesthetic CTR, respectively. There was a significantly higher proportion of CTR hyperesthetic dogs in SCI dogs when compared with control dogs at 6 weeks (*P* = .01), 6 months (*P* = .02), and 1 year (*P* = .001) after surgery. Dogs with hyperesthetic CTR had significantly lower MSTs than did dogs with normal CTR at all 3 time points postoperatively (*P* < .001; Figure 3). All but 3 dogs in the SCI group scored 0 on the SSMS at each time point and therefore no statistical analysis was performed using these data. Two dogs scored 1/4 and one dog 2/4 on the SSMS at the 6‐month and 1‐year postoperative reevaluations, despite owners stating the dogs were comfortable at home. All 3 dogs were classified as having a hyperesthetic CTR at their reevaluation (Video S1).

### 3.4 Open field score in SCI dogs

To determine whether motor score was associated with MSTs, the OFS also was evaluated in 39/40 dogs with SCI at 6 weeks and in 27/40 dogs that returned for reevaluation at 1 year postoperatively. No relationship was found between the OFS at 6 weeks (median, 9; range, 2‐11) and 1 year (median, 11; range, 5‐11) after surgery and the MST at incision and control site in these dogs (*P* = .9).

## 4 DISCUSSION

We demonstrated that paraspinal MSTs can be measured reliably using pressure algometry, with excellent interobserver reliability and acceptable test‐retest reliability in healthy dogs. We investigated and confirmed the hypothesis that dogs with SCI caused by acute TL‐IVDE have lower MSTs at the incision site immediately after a hemilaminectomy compared to healthy dogs, and that the MSTs recover over time. The low MSTs in the early postoperative period are likely caused by nociceptive pain associated with the surgical incision. However, MSTs remained low in a subset of SCI dogs at both the incision site and the control site up to a year after surgery, potentially reflecting development of neuropathic pain. To the best of our knowledge, ours is the first study to evaluate MST of the SCI site over a period of 1 year in dogs with acute TL‐IVDE that underwent hemilaminectomy.

Pressure algometry is considered a reliable tool to evaluate pain in humans^42^, ^43^, ^44^ and although it has been used for research in dogs,^45^, ^46^, ^47^ relatively few studies have evaluated its feasibility and reliability in dogs.^24^, ^25^, ^29^ To evaluate the utility of pressure algometry as an objective means of measuring MST and, by implication, pain, we first evaluated MSTs at 2 different paraspinal sites in 22 healthy Dachshunds. The interscapular region was selected as a control site because this relatively protected site is less mobile, is an unusual location for IVDE and was considered sufficiently distant from the normal sites of IVDE to serve as a useful control. Indeed, we found that MSTs were higher at this site than at the TL junction in healthy dogs. This observation is consistent with previous studies in human and veterinary medicine that have reported different MSTs at different locations because of differences in nociceptor density, distribution, and types, with some regions being highly innervated and having smaller receptive fields compared to others.^25^, ^48^, ^49^ Furthermore, the difference in skin type and amount of overlying soft tissue can contribute to differences in threshold responses.^25^, ^50^, ^51^ We then compared interobserver reliability and found that there was high agreement between observers. In general, pressure algometry measurements are more reliable if performed by a single observer,^26^ but in our study the observers practiced the application of pressure together and their measurements had high consistency.

One study showed that learning avoidance and anticipation may occur in some dogs over time^25^ whereas others found that dogs did not develop local hyperesthesia, learned avoidance, or intolerance.^24^, ^29^ We evaluated test‐retest reliability in neurologically normal healthy dogs tested for 5 consecutive days. No significant difference in MST was found when evaluating the effect of daily algometry, showing that the majority of the dogs tolerated repeated measurements. Our results support the feasibility of repeat testing in this group of dogs. However, the order of testing, anatomical site, site order, time, day, algometry tip diameter, and position of the dog during evaluation all can affect MST measurements and much care should be taken with experimental design.^25^, ^52^

When performing neurological examinations, we noted that some dogs had a strong muscular contraction when performing the CTR even with a very light touch to the skin. Excessive skin rippling or twitching has been described in cats with feline hyperesthesia syndrome. This reaction in affected cats can be spontaneous or elicited by light touch over the lumbar spine and is thought to represent allodynia, which is an abnormal painful response to a normally nonpainful stimulus, such as a light touch.^53^ In people with SCI, allodynia is a frequent symptom of neuropathic pain, reported in almost half of the patients.^1^, ^54^, ^55^ We therefore sought to determine whether there was any relationship between CTR activity and MSTs and categorized the CTR as normal or hyperesthetic in the control and SCI dogs. Overall, 15% of the control dogs had a CTR that was hyperesthetic suggesting this stimulus might be noxious to some healthy dogs.

Despite the use of postoperative analgesics and normal values on the SSMS, the MSTs at the incision site of SCI dogs were significantly decreased when compared with those at the TL site in control dogs for 4 weeks but then returned to normal values. These data likely reflect a normal nociceptive and inflammatory pain response after IVDE and surgery. During the postoperative period, acute pain is expected throughout the inflammatory process and healing phase and can last up to 3 months.^3^ Nociceptive pain occurs when peripheral neural receptors are activated by noxious stimuli such as surgical incisions or trauma, whereas inflammatory pain, a type of nociceptive pain that results from activation and sensitization of receptors by inflammatory mediators, increases gradually from activation of the immune system in response to injury.^56^, ^57^, ^58^ Standard postoperative pain scales were normal at the 7‐, 14‐, and 30‐day time points in our study suggesting that MSTs are more discriminating.

Although the majority of the dogs in our study showed improvement of paraspinal MSTs to normal levels by 6 months after surgery, a subset had persistently decreased MSTs at both the incision and control site compared to healthy controls 6 months and 1 year after surgery. Indeed, because the range of MSTs at the incision site in normal dogs was much wider than at the control site, more SCI dogs were identified with MSTs below the minimum in group 1 dogs at the control site, with nearly 33% of dogs falling below the reference range at 6 months and 15% at 12 months. Moreover, low paraspinal MSTs were associated with hyperesthetic CTRs in these dogs. These persistently low thresholds might reflect the presence of SCI‐associated neuropathic pain in dogs, similar to the phenomenon that occurs in people,^8^, ^18^ but in a lower proportion of patients. Despite these findings, as illustrated in Video S1, the owners of these dogs did not report any signs of pain at home. Because signs of chronic pain in dogs often are subtle, it is likely that neuropathic pain is underrecognized in veterinary medicine.^59^ Additional work should be performed to determined how important a problem it is and whether it affects QOL for affected dogs.

One limitation of our study is that the observer was not blinded to the groups (SCI versus neurologically normal healthy dogs). However, in attempting to minimize potential bias, the observer operating the algometer did not look at the algometer scale as they performed the test. All dogs that had undergone surgery received a tapering course of analgesic drugs for 2 weeks postoperatively, which could have affected the MST measurements reported for days 7 and 14. However, it would have been unethical to withhold appropriate analgesic drugs. Moreover, despite implementation of pain management, SCI dogs still had low MST emphasizing the need for postoperative analgesia for at least 2 weeks. In fact, our data show lower MST values for at least 42 days postoperatively in the SCI dogs compared to control dogs (group 1). This observation might indicate that analgesia should be provided for a longer period after surgery, although owners did not report any appreciable pain and it is unknown at what MST threshold analgesia should be provided. Another potential limitation of our study is that the MSTs of sites below the lesion were not evaluated. Spinal cord injury patients may develop signs of neuropathic pain not only at the level of the injury, but also above and below the lesion.^13^, ^60^ We evaluated the level of the injury and areas above the injury. Although investigating MSTs below the level of the injury is important, it was difficult to identify a region in every dog that was not in some way involved with the surgical incision or within a short distance of the site of injury. Future studies on pain below the level of the injury might be indicated after the surgical incision heals, particularly because such neuropathic pain often becomes evident only in the chronic phase of injury.^4^, ^13^ Finally, despite efforts to minimize any behavioral influence, assessing MST in animals still can be challenging. Differences in individual temperament may affect the dog's response to pressure algometry, and some studies have shown that hyperactive dogs also appear less willing to cooperate with MST testing, leading to difficulties in data collection.^24^

We determined that pressure algometry can be used to establish paraspinal MSTs reliably in pet dogs at the level of SCI and cranial to that region and that MSTs vary depending on the paraspinal location being tested. We found that dogs with SCI that have undergone decompressive surgery have decreased paraspinal MST in the early postoperative period, but this threshold normalizes in most dogs by 6 months after surgery. However, between 15% and 33% of SCI, dogs may develop chronic neuropathic pain reflected by low paraspinal MST at the control site and hyperesthetic CTR. Improving long‐term assessment in SCI dogs is important in order to identify dogs with persistent chronic pain. Chronic pain may affect the dog's QOL and recognizing this type of pain is essential for making the best treatment decision and advising owners.

## CONFLICT OF INTEREST DECLARATION

Authors declare no conflict of interest.

## OFF‐LABEL ANTIMICROBIAL DECLARATION

Authors declare no off‐label use of antimicrobials.

## INSTITUTIONAL ANIMAL CARE AND USE COMMITTEE (IACUC) OR OTHER APPROVAL DECLARATION

This study was approved by the North Carolina State University IACUC. Informed consent was obtained from all owners.

## HUMAN ETHICS APPROVAL DECLARATION

Authors declare human ethics approval was not needed for this study.

## Supporting information


**Supplementary Video 1** Video shows CTR evaluation and spinal palpation in a SCI dog.
