# Supplementary material for: Long‐term postoperative pain evaluation in dogs with thoracolumbar intervertebral disk herniation after hemilaminectomy
Source: J Vet Intern Med. 2020 May 28;34(4):1547–55. doi: 10.1111/jvim.15800 (PMC7379041; doi:10.1111/jvim.15800)
Supplement: Supplementary file 1 — Supplementary Video 1 Video shows CTR evaluation and spinal palpation in a SCI dog. [file JVIM-34-1547-s001.docx]

**Supplementary Video 1: Video shows CTR evaluation and spinal palpation in a SCI dog.**

The hyperesthetic CTR is characterized by a strong reflex contraction of the cutaneous trunci muscle in response to pinching with the hemostatic forceps. In addition, this dog showed a strong aversive behavioral response to even light touch to the skin.
